# Implementation and acceptability of strategies instituted for engaging men in family planning services in Kibaha district, Tanzania

**DOI:** 10.1186/s12978-016-0253-6

**Published:** 2016-11-21

**Authors:** Judith Msovela, Anna Tengia–Kessy

**Affiliations:** 1 National Institute for Medical Research (NIMR), P.O. Box 9653, Dar es Salaam, Tanzania; 2 Muhimbili University of Health and Allied Sciences (MUHAS), P.O. Box 65015, Dar es Salaam, Tanzania

**Keywords:** Acceptability, Strategies, Engaging men, Family planning, Kibaha

## Abstract

**Background:**

Men as the main decision makers in most of African families have an important role to play towards acceptance of family planning methods. This study sought to identify strategies used to engage men in family planning services and determine the extent to which men in Kibaha district in Tanzania accept these interventions.

**Methods:**

We conducted a cross sectional study using both quantitative and qualitative techniques. We used a questionnaire to interview a random sample of 365 of currently married or cohabiting men who had at least one child under the age of five years. We further conducted in-depth interviews with health workers involved in delivering reproductive health services as well as community dispensers of family planning commodities. Descriptive analysis was used to determine the extent to which men were engaged in family planning services. The data from the indepth interviews were analysed manually according to the predetermined themes, guided by the grounded theory to identify the existing strategies used to encourage male involvement in family planning services.

**Results:**

According to the key informants, strategies that are used to encourage men to engage in family planning services include invitations through their spouses, either verbally or by using partner notification cards, incorporating family planning messages during monthly meetings and community outreach reproductive health programs. Of 365 men responding to the questionnaire, only 31 (8.4%) said they were invited to accompany their spouses to family planning clinics. Among them, 71% (22/31) visited family planning clinics. A third (32%) of the respondents had heard of community health meetings and only 20.7% of them attended these meetings. More than a third (12/34) of men who attended these meeting asserted that family planning messages targeting men featured in the agenda and subsequently half of them visited health facilities for family planning services.

**Conclusions:**

Existing strategies such as invitations to clinics and community sensitization have shown to encourage men to engage in family planning services. However, these interventions reach few men and hence there is a need to rolling them up to improve uptake of family planning services.

**Electronic supplementary material:**

The online version of this article (doi:10.1186/s12978-016-0253-6) contains supplementary material, which is available to authorized users.

## Plain English summary

Family planning allows couples to have the number of children they want and to decide intervals between pregnancies through the use of contraceptive methods. Men in Tanzania, as in most African countries influence the use of family planning methods as main decision makers. This study sought to document implementation and acceptability of approaches used to engage men in family planning services in Kibaha district in Tanzania.

We interviewed 365 men living with female partners and had at least one child under the age of five years. We also interviewed health workers involved in delivering reproductive health services to identify approaches used in engaging men in family planning services.

The main approaches identified were: invitations to attend reproductive health clinics through their spouses, either verbally or through notification cards, delivering family planning messages in community meetings, and outreach reproductive health programs. Of the 365 men interviewed, 31 said they were invited to accompany their spouses to family planning clinics and 22 of them visited a clinic. Of 117 men who had heard of community health meetings, 34 of them attended these meetings. Twelve of them affirmed that family planning messages targeting men featured in the agenda and subsequently six men visited a health facility for family planning services.

In conclusion; the approaches used have shown to encourage men to engage in family planning services. However, these interventions reach a few men and hence there need to be scaled up to improve acceptance of the services.

## Background

Men as the main decision makers in most of African families have an important role to play towards acceptance of family planning (FP) methods scientifically recommended and approved by the World Health Organization (WHO) as well as the Ministry of Health (MoH) in a given country. Men can engage in FP services either as service users or as supporters to their female partners. Various studies globally have reported that more than 90% of men have general knowledge of FP [1–3]. However, it is established that men lack specific knowledge on different methods from which they can choose, how to access services, and how the different methods work [2, 4–7]. This knowledge gap among men in Tanzania contributes to limiting their chances of accepting FP, both as users and as supporters [2, 8]. Low male acceptance of FP has been associated with low prevalence of contraceptive use in Tanzania [9]. Reproductive health (RH) interventions targeting both men and women may help to increase the knowledge on, and acceptability of, FP services. Men’s participation in RH education programs has positive effects, not only on RH but also on the general health seeking behavior of their female partners [10]. Research findings have established that, in the absence of direct discussion, women often assume (and sometimes incorrectly) that their male partners are against the use of FP [11]. Therefore, programs involving men may improve their knowledge, intra spousal communication, enhance gender equitable attitudes and increase FP use and continuation [12, 13].

Despite the recognized role of involving men in RH services, programs targeting them in many countries do not seem to be scaled-up. Consequently, many men do not see the need for their involvement in such services [13].

The Tanzanian government has policy and strategy documents that integrate men involvement in RH issues, including access to and adoption of FP services [8, 14, 15]. Nevertheless, there is an evidence gap on the implementation of these policies and strategies aimed at encouraging men participation in their personal capacity as users and as supportive partners. Men may participate by visiting RH clinics to access FP education, male-based methods, counseling and HIV testing. This study aimed to identify existing strategies aimed at engaging men in FP issues, document the extent to which such strategies have reached them and their responses in Kibaha district, Pwani region, Tanzania.

## Methods

We conducted this mixed method design study in Kibaha district of Coastal region in Tanzania. The district consists of 11 wards which are geographically defined administrative localities formed by at least three villages. Each village constitutes several hamlets (neighbourhoods) if the area is rural or streets if the area is urban [16]. According to the most recent national population census, Kibaha district has a total population of 75,899, out of which15,598 are adult males [17]. The health care delivery structure comprises of a public health centre and 23 dispensaries of which 16 are government (public). Family planning services are available at the public health facilities only.

Respondents were selected through a multistage stratified sampling technique. The 11 wards of the district were stratified into rural (8) and urban (3) to have both representations since availability of health services usually differs between the two strata. From each stratum, one ward was randomly selected, namely Soga and Mlandizi to represent rural and urban settings respectively. At the second stage, a simple random method was used to select five villages from the two wards, proportionately to their sizes. Hence three out of 10 villages were selected from Mlandizi ward and two out of five villages were selected from Soga ward.

From each village, the list of men who met the eligibility criteria was prepared. The eligibility criteria were all men aged between 18 and 60 years, who were living with their spouses and had children under the age of five years. These lists were prepared with the assistance of either the respective village chairpersons or community health workers (CHW) who are usually very familiar with the local settings. A systematic random selection method was used to select respondents from each village, proportional to the number of eligible men listed. The sample size for the main study (unpublished) was obtained using the formula for single proportion [18] considering a prevalence of contraceptive use among couples of 38.3% in the region [2], at 95% confidence level and margin of error of 5%, yielding a sample size of 365 men.

A pre-designed questionnaire was used to collect data on sociodemographic characteristics such as age, occupation, level of education and number of living children. We also collected information on respondent’s knowledge and use of family planning services; whether a respondent visited a health facility for FP information and commodities and whether he had ever been invited to health facility for FP services as well as whether he ever attended a community meeting where FP issues were discussed. Respondents were mostly interviewed at their homesteads and only two of the selected men could not be traced for interviews. These were replaced by selecting two other men randomly from the list.

We also selected eight key informants (KIs) purposively, based on their involvement in FP service delivery for in-depth interviews (IDI). They included the district reproductive and child health (RCH) coordinator, in-charge of Mlandizi RCH clinic and two health care providers working in the RCH clinics at the Mlandizi and Soga dispensary. Two community-based distributors of FP commodities also participated in IDI. The aim of the KIs interviews was to gather information on the key interventions in place to encourage men engagement in FP services. The KIs interviews also enquired about men acceptance and use of family planning services, either as clients or as supporting partners; and the opinion of the KIs on the influence of the existing strategies in increasing male involvement in FP issues. All KIs consented for tape-recording that allowed verbatim transcriptions later on.

The quantitative data were checked for clarity and accuracy immediately after their collection. Two independent data entrants entered the data in EpiData version 3.1software. The data were then cleaned and subsequently exported to STATA version 11.2 statistical package for analysis. This study was mainly exploratory therefore simple descriptive analysis was done to describe the magnitude of reach of various strategies used to engage men in FP services and the extent to which men responded to those strategies.

The qualitative data was transcribed verbatim and thereafter coded and arranged thematically according to the research objectives, focusing on contents and patterns of the facts presented [19]. The key issues described included the strategies used and their acceptability in encouraging men to engage in family planning services. A social scientist was responsible for identifying key points whose quotes were used to enrich the presented data.

## Results

### Sociodemographic characteristics of the respondents

The study involved 365 men (239 from Mlandizi and 126 from Soga wards) and eight key informants (KIs) directly responsible for providing RCH services. The mean age of the men interviewed was 35 ± 9.25 years and the highest proportion (43.8%) was aged between 28 and 37 years (Table 1). The highest level of formal education attained by most respondents (65.5%) was primary education and only 74 of them (2.5%) had accessed secondary school education. About two-fifth (41.1%) of the respondents were involved in the agricultural sector, mainly as informal small farm-holders commonly known as peasants.

**Table 1:** Sociodemographic characteristics (*N* = 365)

Characteristic	Number	Percentage
Age group in years		
18–27	71	19.4
28–37	160	43.8
38–47	90	24.7
48+	44	12.0
Level of education:		
No formal education	31	8.5
Primary school incomplete	21	5.8
Primary school complete	239	65.5
Secondary school and more	74	2.5
Occupation:		
Subsistence farmers	152	41.2
Employed	57	15.6
Small business	72	19.7
Laborers	84	23.0

Of the eight key informants (KIs), half were registered nurse midwives while the remaining comprised of a clinical officer, a public health nurse and two-trained community-based distributors of FP commodities. The duration of their involvement in RCH provision ranged between two and 30 years.

### Strategies used to involve men in family planning at health facility level

According to the KIs, there are three main strategies used to increase male participation in RH services delivered at health facilities. These are giving couples the first priority for services, encouraging women to bring along their spouses during subsequent visits and early issuing of delivery packs to accompanied antenatal women.

Another strategy is that service providers ask women to invite their spouses either verbally or by using partner notification cards. This approach seemed to motivate the health workers who felt that both clients and their spouses valued their advice as quoted saying:

*“When you see a woman accompanied by her partner, you are encouraged because you realize that the strategies in place are working and we give service priority to such women”* (KI, Soga dispensary).

Some KIs however claimed that some of the men are not reachable via the partner notification cards since some women never present these cards or show them rather late to allow planning for the intended visit. As argued, women sometimes hesitate to deliver the messages to their spouses as they simply assume that they would not accept the offer as lamented by one of the KIs:

*“You instruct a woman to ask her spouse to accompany her in the next visit, but she immediately responds that her spouse is not around or she is not sure if he would agree”* (KI, Mlandizi HC).

The strategy of issuing delivery packs to accompanied women during antenatal visits assumes that women will press their spouses to accompany them in order to seize the opportunity, while at the same time accessing FP information directly from health workers. This would additionally relieve women the tension they get at home when persuading their spouses with their invitation cards. KIs were convinced that incorporating FP education messages in the antenatal care service package, coupled with persuading men to escort their spouses to clinics is an effective men-targeting strategy. Men would be coming to follow the progress of the pregnancies of their spouses but eventually receive FP information that is helpful to their family development. The following remark from a dispensary health worker refers:

*“Delivery packs are given to pregnant women at 28 weeks (early) if accompanied by spouses whereas the packs are given at 36 weeks (late) for unaccompanied women* (KI, Soga dispensary).

### Community-level strategies for involving men in family planning

Key informants additionally reported various strategies implemented at the community level to encourage male participation in RH. These included incorporating FP messages during monthly meetings and community outreach RH programs conducted jointly by the local leadership and health facilities. Provision of RH education to the community is an example of an outreach program pointed out during KI interviews. The program is geared towards sensitizing youth who are in reproductive age about the importance of male involvement in RH services*.* From each ward, 20 youths undergo training on RH issues and on how to sensitize men in order to increase their involvement in RH services. Experience during one of the trainings revealed that most of the participants had little knowledge of RH issues, as testified by one of the respondents who was quoted saying:

*“It has been observed in one of the training sessions that, most men did not have knowledge on RH services and why they should be involved in the services* (KI, Mlandizi Health Centre).

There are other various community-level initiatives to ensure male involvement in RH issues and in particular FP services. For example, in each village, there are two community health workers (CHWs) or community based distributors (CBDs), a male and a female. They provide services that include maintaining a register of all households, providing FP education in homes, distributing pills and condoms and referring clients who prefer other methods to clinics. They also distribute leaflets in their catchment areas and conduct house-to-house visits to provide counseling on FP. They use the same opportunity to target married men although the visits seem to target specific types of couples as expressed by one of them:

*“I mainly do home visits to clients who have many children and couples who seem to have difficulties in accepting FP…for example if a woman wants to use FP but her spouse denies her, I ask him to come to the village office or I go to his home”* (KI, Soga village)

According to the key informants, additional strategies for male involvement in FP issues included incorporating FP services in the bi-annual national campaigns such as immunization and vitamin A supplementation days. Integrated services are mainly counseling, health education and distribution of FP commodities. Additionally, each village has an annual village health day and the community-based service providers utilize the opportunity to provide FP services. Since both the campaigns and the village health days are well attended by men, these provide good avenues for sensitizing them and scaling-up FP services.

Other reported techniques included the use of drama at the ward level in which drama groups usually perform various shows related to RH on quarterly basis. To motivate the community to attend and receive the intended FP messages, children under the age of five years who performed well in terms of gaining weight and completing vaccination schedules are often given rewards such as mosquito nets.

Further efforts include sensitizing religious leaders from various denominations on the importance of male involvement in RH services. This is an entry point for disseminating FP messages to their adherents through preaching as revealed in one of the interview sessions:

*“We conducted a meeting with religious leaders to sensitize them about male involvement in RH services despite the challenges and sometimes misconceptions especially about using FP methods. The importance of FP in overcoming the current economic hardships was discussed and they seemed to understand”* (KI, Soga village).

In view of the increasing awareness and the need for involving men in RH services, key informants additionally proposed provision of seminars to community leaders as well as collaboration between various actors for sustainability of the trainings at community level. Some respondents were of the opinion of establishing an Act to compel reluctant or conservative men accompany their partners to FP service delivery points:

*“I recommend that an Act be formulated to make sure that all men accompany their spouses to RH services”* (KI, Mlandizi, Health Centre)

However, some KIs had reservations with enacting an Act as it might take long time, and it might not sound realistic to many people. The KIs additionally suggested the use of peer educators to approach men and motivate them by giving some gifts when they escort their spouses to RCH clinics. Another approach suggested was establishing local radios that incorporate sessions focusing on male involvement in RH issues in their programs. KIs went further into suggesting provision of health education through video shows in all health facility-waiting lounges where clients /patients hang around for services:

*“Videos should be available in all waiting lounges and display various messages of FP so that clients/patients get education as health workers are not always adequate to provide the necessary education”* (KI, Mlandizi Health Centre).

### Extent to which strategies for encouraging men use FP services reach them

To understand to what extent some of the strategies aiming at encouraging men in FP services implemented at health facility and community levels reach men and their response to such moves, we asked all the surveyed men if they had ever been invited to health facilities for FP information and services; and if they had ever attended community meetings which incorporated FP issues in the agenda. Data revealed that inviting men to attend FP clinics with their spouses was a common practice although only 31 (8.4%) of the men acknowledged receiving such invitations either verbally 23 (74.2%) or via partner notification cards 8 (25.8%). Majority of men (22/31, 71.0%), who reported to ever been invited to health facilities for FP services, were from Mlandizi ward. Overall, 22 (71.0%) of invited men responded by visiting the clinic, whereas the rest claimed to be preoccupied with other chores. Higher proportion of men from Soga (77.8%) responded to the invitations than Mlandizi (68.2%). More than 50% of men in all age groups reported to have visited health facilities in response to the invitation while all the five men aged 48 years and above as well as the two men who did not complete primary education responded positively. Regarding occupation, laborers accepted the invitations least (37.5%) compared to the other occupation categories (Table 2).

**Table 2:** Men exposure and their response to invitations for FP services provided at health facilities

Characteristics	Invited to Health facility for FP services	Accepted the invitations
	Number (%)	Number (%)
Residence		
Mlandizi	22(71)	15(68.2)
Soga	9(29)	7(77.8)
Age group in years		
18–27	6(19.4)	4(66.7)
28–37	13(42.0)	9(69.2)
38–47	7(22.6)	4(57.1)
48+	5(16.1)	5(100)
Level of education		
Primary school incomplete	2(6.5)	2(100)
Primary school complete	17(54.8)	12(70.6)
Secondary school and more	12(38.7)	8(66.7)
Occupation		
Subsistence farmers	10(32.3)	7(70.0)
Employed	6(19.4)	5(83.3)
Small business	7(22.6)	7(100)
Laborers	8(25.8)	3(37.5)

A third (32%, 117/365) of all surveyed men had heard of a health meeting in their communities. However, Soga ward had a relatively higher proportion of men (36.5%, 46/126) who reported to have ever heard about health meetings happening in their community as compared to those from Mlandizi (29.7%, 71/239). Among respondents who had ever heard about health meetings in their communities, 29.0% (34 of 117) confirmed to have attended such meetings (Table 3). Less than half (41.1%, 14/34) of men who attended the village health meetings said that FP was one of the agendas discussed, and six of them consequently visited a health facility for FP service in supporting their partners. When enquired whether there were any FP messages targeting men at the meetings they attended, two thirds of them (9/14) responded affirmatively. They asserted that the messages carried advice for men to value the importance of accompanying their spouses to FP clinics, and either use or support spouses in using the FP methods. Five of the nine men who heard of FP messages in the meetings subsequently visited clinics to access the services.

**Table 3:** Men exposure to FP messages in health meetings at community level

Characteristics	Number	Percentage
Awareness of Health meeting (*n* = 365)		
Aware	117	32.1
Attendance to the meeting (*n* = 117^a^)		
Attended	34	29.1
FP topic discussed (*n* = 34^b^)		
Yes	14	41.2
FP messages targeting men (*n* = 14^c^)		
Yes	9	64.3

^a^Ever heard of a community meeting, ^b^Attended a community meeting, ^c^Attended a community meeting where FP was one of the agendas

Respondents provided opinions regarding additional strategies to improve male involvement in FP services. Most (90%) of them were in favor of more sensitization and health education in order to clear their doubts and misconceptions about FP issues promoted in the health service sector. They further proposed for service providers to visit them at their homes or working places and social gathering areas where they are mostly found and distribute FP leaflets and brochures.

## Discussion

It is widely argued that men in most traditional African cultures play an important role in influencing household health seeking behavior, including accepting and approving the use of FP services. Available evidence demonstrates that although male involvement in FP increases women’s uptake and continuation of use of the methods, participation of men in supporting use of, and personally using the methods has remained low in developing countries [7].

Findings from this study show existence of various strategies at different levels aimed at encouraging men to accompany their partners to health facilities for FP services. The strategies employed by health providers include giving couples the first priority for services. This strategy is also implemented in some countries such as Kenya, and it has shown to encourage men to accompany their spouses in reproductive health services [20]. Encouraging women either verbally or through notification cards to bring along their spouses during subsequent visits was reported to be used to encourage men to visit health facilities. Similar strategies were documented in other countries in sub Saharan Africa such as Malawi and Uganda [21, 22]. Furthermore, incentives such as early issuing of delivery packs to accompanied antenatal mothers was used to encourage women to press their spouses to accompany them to the antenatal clinic (ANC) services. ANC incorporated FP issues and therefore, when men visit the clinic they get an opportunity for accessing information and are also sensitized on FP issues.

The present study indicates incorporation of FP in various community-level activities such as meetings, outreach programs and sensitization of community and religious leaders. These initiatives have been implemented in countries such as Malawi and Kenya and have shown to encourage men to engage in reproductive health issues [23, 24]. As the findings from men interviews reveal, the existing strategies do not reach most of them. For example, only 8.4% of all the interviewed men had ever been invited to the nearest health facilities for FP services. However out of the few who were invited, 71% of them visited health facilities for FP services. One of the reasons for this finding could be that, invitations, especially through notification cards were given to men who had competing activities at the same time. This strategy also might have depended on the perceptions and attitudes of HWs towards male involvement and therefore their readiness to give the right and full packaged information constrained in some ways. That is to say, those who might have negative attitudes towards male use of FP methods might not have seen the importance of taking extra efforts of giving the women they attended enough information to feed their spouses, including encouraging these men to accompany them to the clinics [23].

Passing the information about HWs’ invitation of their partners to the clinics might have posed another challenge to women who for a long time had experienced inadequate spousal communication in societies where males dominate decisions and have little time to stay with and listen to their women. Nevertheless, this argument is challenged when one considers the encouraging number of the men who after getting invitation responded positively and visited the clinics. The reported level of response to the invitations is in line with various studies in Africa which have demonstrated that use of notification cards or invitation letters increased men attendance to clinics for sexually transmitted infections (STIs) and prevention of mother to child transmission (PMTCT) of HIV infections [21, 25–27]. There are good lessons that could be learned from men in this study and in particular those who after receiving invitations decided to accompany their female partners to the clinics. This suggests that a sustained use of this strategy and in a more tactical manner could help promote the FP practices traditionally concentrated to women to a larger number of married or engaged men.

The present study reveals that although health meetings occur regularly in the communities, only a third of men were aware of such meetings that translated to low attendance. If attendance to the meetings would be on compulsory basis by announcing to the general public, that might probably work more effectively. However, this done that way would affect people’s mindset beforehand and those who might attend would not value the messages given as they would feel forced by the government system to attend and might be considered a political affair. Moreover, the present study findings show that only one third of the meetings incorporated FP as one of the agendas and had messages that targeted men. Interestingly, a higher proportion of men in this study, who ever attended health meetings, visited health facility for FP services as compared to their counterparts who did not attend such meetings. Health meetings also serve as avenues for sensitizing men to engage themselves either as clients or supportive partners in RH issues/services. Therefore, the meetings provide opportunities for education on RH and clear myths and misconceptions about modern FP methods. Such misconceptions have been associated with men disapproving their partners to use the methods [28, 29]. Provision of FP education through community meetings has shown to be effective in reaching men in various parts of the world such as Cambodia, Egypt and South Africa [29, 30]. From these lessons learnt, it is evident that if these community interventions are consistently used will increase male participation in RH services.

Besides the existing strategies, the interviewed men proposed more efforts to increase male involvement in RH services since the existing strategies do not reach majority of men. Reaching men at their workplaces or in men-based social gathering places are some of the proposed approaches. Evidence shows that men receive information about RH issues through their peers usually at working and their social gathering places. Based on this reality, men could be reached at such places to increase access to FP services particularly education since they do not frequently visit health facilities and are not usually available at home during the day. Experience from Nigeria as reported by Akindele and Adebimpe [31] reveals use of various avenues to reach men to have been successful. For example, a FP program in Kenya successfully used workplace motivators who provided information about contraception and commodities and referred men to clinics for vasectomy [32].

Although the findings from this study have provided the current situation of existing strategies for increasing male engagement in family planning services there are some methodological limitations. Taking into consideration the large number of districts in the country and their diversity in both social and administrative issues, findings from one district might be context specific and may not reflect the situation in Tanzania as a whole. However, the use of mixed methods for data collection has provided deep understating of the strategies used in encouraging men to engage in FP services and the same approaches could be implemented in other settings similar to the study district.

## Conclusions

The study establishes the existence of strategies to encourage men to use FP services in the study area. Men invitation to health facilities through their female partners either verbally or using notification cards and community sensitization activities are some of the interventions that have been used. These strategies have shown to encourage men engagement in reproductive health issues. However, the interventions do not reach most of them. Therefore, there is a need to increase the coverage of the interventions to improve uptake and continuation of FP.

## Additional file

Additional file 1: Dataset of Men's Interviews on Male Involvement in Family Planning Services. (XLSX 24 kb)

## Abbreviations

ANC: Antenatal care
CHW: Community health workers
FP: Family planning
HW: Health worker
KI: Key informant
MUHAS: Muhimbili University of Health and Allied Sciences
NBS: National Bureau of Statistics
PMTCT: Prevention of mother to child transmission
RH: Reproductive health
STIs: Sexually transmitted infections
